# Pituitary adenomas and cerebrovascular disease: A review on pathophysiology, prevalence, and treatment

**DOI:** 10.3389/fendo.2022.1064216

**Published:** 2022-12-13

**Authors:** Robert C. Osorio, Jun Y. Oh, Nikita Choudhary, Meeki Lad, Luis Savastano, Manish K. Aghi

**Affiliations:** Department of Neurological Surgery, University of California, San Francisco, San Francisco, CA, United States

**Keywords:** pituitary adenoma (PA), cerebrovascular disease (CVD), cerebral infarct, stroke, pituitary neuroendocrine tumor (PitNET)

## Abstract

Pituitary adenomas (PAs) have been shown to cause excess cardiovascular disease comorbidity and mortality. Cerebrovascular disease (CeVD) is a small subset of cardiovascular disease with high morbidity, and its risk in patients with pituitary adenomas has been sparingly explored. In this review, we examine what is known about the prevalence of cerebrovascular disease in patients with PAs, from its initial discovery in 1970 to present. An abundance of literature describes increased cerebrovascular mortality in patients with acromegaly, while research on other PA subtypes is less frequent but shows a similarly elevated CeVD mortality relative to healthy populations. We also review how cerebrovascular risk changes after PAs are treated, with PA treatment appearing to prevent further accumulation of cerebrovascular risk without reversing prior elevations. While acromegaly-associated CeVD appears to be caused by elevated growth hormone (GH) levels and Cushing disease’s elevated glucocorticoids similarly cause durable alterations in cerebrovascular structure and function, less is known about the mechanisms behind CeVD in other PA subpopulations. Proposed pathophysiologies include growth hormone deficiency inducing vessel wall damage or other hormone deficits causing increased atherosclerotic disease. Early diagnosis and treatment of PAs may be the key to minimizing lifetime CeVD risk elevations. More research is needed to better understand the mechanisms behind the increased CeVD seen in patients with PAs. Physicians caring for PA patients must remain vigilant for signs and symptoms of cerebrovascular disease in this patient population.

## Introduction

Pituitary adenomas (PAs) are benign tumors arising within the anterior lobe of the pituitary gland. After glioma and meningioma, they are the third most common primary brain tumor and compose roughly 15% of all intracranial tumors (1–3). They are subclassified by their ability to secrete hormones of the anterior hypothalamic-pituitary axis. Functional adenomas cause hormonal hypersecretion, leading to significant symptoms such as acromegaly, galactorrhea, hypogonadism, or Cushing disease. In contrast, nonfunctional adenomas (NFPAs) are considered “silent” and do not secrete hormones at clinical levels. NFPAs comprise 15-35% of all PAs (4–7) and may cause mass effects such as headache, hypopituitarism, or visual deficit (6, 8–10). Meanwhile, other NFPAs cause no symptoms and instead are discovered incidentally on neuroimaging studies (11).

While much attention is given to the common presentations of PAs, they have also been associated with other systemic comorbidities such as cardiovascular disease. Cardiovascular disease is a diagnostic term encompassing disease processes related to compromised heart structure and function, uncontrolled hypertension, and atherosclerosis, including heart failure, myocardial ischemia, arrhythmias, valvopathies, and sequelae of peripheral arterial disease, including limb ischemia and stroke (12). This is not unique to PA patients, as cardiovascular disease and its sequelae are the leading cause of noncancer death in all brain tumor patients (13). Due to the benign nature of PAs, however, cardiovascular disease is the leading cause of death in PA patients, accounting for 40% of all deaths (14). While most notable in cases of acromegaly and Cushing disease, both patients with functional and nonfunctional pituitary adenomas have been shown to carry an increased risk for cardiovascular disease comorbidity and mortality (15–17).

This review article examines one common and particularly dangerous subset of cardiovascular disease in PA patients, cerebrovascular disease (CeVD). CeVD is defined as the set of diseases causing cerebral ischemia or hemorrhage, and includes stroke, carotid vertebral or intracranial stenosis, aneurysms, and vascular malformations (18). Among the abundance of literature reporting the cardiovascular morbidity and mortality associated with PAs, only a small subset examines CeVD separately, with fewer still comparing the disease’s incidence and mortality between PA subtypes. This paper summarizes what is currently known regarding the pathophysiology, risk factors, epidemiology, mortality, treatment, and prevention of CeVD in patients with PAs. We also examine what is known about how these factors vary depending on both the type of adenoma as well as the sex of the patient, which may affect both the hypothalamic-pituitary (HP) axis and cardiovascular/cerebrovascular risk.

## Methods

A comprehensive literature review was conducted using PubMed’s online repository of published articles. Searches were conducted using the keywords “pituitary adenoma”, “pituitary neuroendocrine tumor”, “acromegaly”, and “Cushing disease”, with each keyword paired with combinations of “cerebrovascular disease”, “cerebrovascular outcomes”, “stroke”, “cardiovascular disease”, “atherosclerosis”, and “vascular disease”. Resulting articles from these queries underwent abstract review. Studies that reported data on cerebrovascular disease incidence, prevalence, risk factors, pathophysiology, or treatment in patients with pituitary adenomas were subsequently reviewed in their entirety. Exclusion criteria included non-English studies, and studies that primarily focused on the effects of radiation therapy on cerebrovascular disease.

## Prevalence of CeVD morbidity and mortality in PA patients

Previous studies have shown the elevated risk of CeVD in a broader cohort of all PA patients. A 1999 UK cohort study examined 331 patients with PAs and found a significant increase in ischemic stroke incidence relative to the general population (19), a finding corroborated by a more recent nationwide analysis as well (20). In contrast, most studies focus on specific cohorts of PA patients, with each study often examining a different subpopulation. Because of this variety, it is difficult to compare CeVD morbidity and mortality between PA subtypes. Nevertheless, increased CeVD morbidity and mortality has been shown in a number of differing PA populations, and are subsequently reported below by the group examined.

### CeVD in PA patients with acromegaly

The most common subpopulation examined in PA patients is acromegaly, a disease that carries an incidence of 2-11 cases per million person-years and a prevalence of 28-137 per million patients (21, 22). Cohort studies have found that CeVD is responsible for 3.2-15.4% of deaths among these patients (**Table 1**) **(** 23–30). Excess mortality relative to the general population is often reported as standardized mortality ratio (SMR), defined as deaths observed in a patient cohort relative to expected number of deaths in an age and sex-matched healthy population. In examining how these numbers vary in PA patients there are eight reported studies that estimate CeVD SMRs for patients with Acromegaly.

**Table 1:** Cerebrovascular outcomes related to PA patients with acromegaly.

Authors and Year	No. of Patients	Population Examined	Main Findings
Wright et al., 1970 (23)	194	Acromegaly	Eight patients (4.1%) died from CeVD. CeVD mortality was significantly elevated in women but not in men.
Alexander et al., 1980 (24)	164	Acromegaly	14 patients (8.5%) died from CeVD. CeVD SMR in men: 10 CeVD SMR in women: 7.7
Bengtsson et al., 1988 (25)	166	Acromegaly	11 patients (6.6%) died from CeVD. Vascular disease was a significant cause of excess death in all patients (CeVD was not separately assessed).
Rajasoorya et al., 1994 (26)	151	Acromegaly	5 patients (3.3%) died from CeVD. * Overall CeVD SMR: 3.3
Orme et al., 1998 (27)	1,362	Acromegaly	44 patients (3.2%) died from CeVD. Overall CeVD SMR: 2.06 CeVD mortality was significantly elevated in patients under 35 years old. CeVD mortality increased with longer duration of acromegaly, while cardiovascular disease mortality did not. CeVD mortality was not affected by post-treatment GH levels, while cardiovascular disease mortality was. CeVD mortality was higher in younger populations, while cardiovascular disease mortality was not.
Ayuk et al., 2004 (28)	419	Acromegaly	20 patients (4.8%) died from CeVD. Overall CeVD SMR: 2.68 Tumor size and extension did not affect all-cause mortality (CeVD not separately assessed).
Kauppinen-Mäkelin et al., 2005 (29)	334	Acromegaly	8 patients (2.4%) died from CeVD. * CeVD SMR was not assessed.
Sherlock et al., 2009 (30)	501	Acromegaly	77 patients (15.4%) died from CeVD. Overall CeVD SMR: 2.7
Esposito et al., 2018 (31)	1,089	Acromegaly	Overall CeVD SMR: 3.99
Oh et al., 2021 (20)	31,983; 19,416 NFPAs (4,077 with hypopituitarism); 8,871 prolactinomas; 2,613 GH-secreting adenomas; 1,083 ACTH/TSH-secreting adenomas.	All PAs	Ischemic stroke SIR in patients with GH adenomas: 1.83 Hemorrhagic SIR in patients with GH adenomas: 2.16 Ischemic stroke mortality HR in patients with GH adenomas: 1.38 Hemorrhagic stroke mortality HR in patients with GH adenomas: 1.37

CeVD, cerebrovascular disease; SMR, standardized mortality ratio; GH, growth hormone; SIR, standardized incidence ratio; HR, hazard ratio.

The first study to examine the incidence of CeVD mortality in acromegaly patients was published by Wright et al. in 1970, which found an elevated risk of CeVD mortality in women but not men (23). Ten years later, Alexander et al. examined 164 patients with acromegaly and found CeVD mortality to be significantly elevated in all patients regardless of sex (male SMR 10, female SMR 7.7) (24). Although less prominent, Rajasoorya et al. confirmed these findings in 1994 in 151 acromegalic patients (overall SMR 3.3) (26). Orme et al. was the first study to examine the rates of CeVD mortality in a larger acromegaly analysis (27). Among 1,362 acromegaly patients, 44 (3.2%) died from CeVD, corresponding to a standardized mortality ratio of 2.06. This study was followed by Ayuk et al. in 2004, Sherlock et al. in 2009, and Esposito et al. in 2018, all of which also reported increased CeVD mortality in acromegaly (SMR 2.68, 2.70, and 3.99 relative to healthy non-acromegalic populations, respectively).

The most recent study on cerebrovascular mortality also happens to be the largest. In 2021, Oh et al. conducted a nationwide study in Korea, examining 31,983 patients with PAs over ten years by examining health insurance databases and the country’s Rare Intractable Disease database, where all patients with PAs are registered upon diagnosis. They examined the standardized incidence ratios for both ischemic and hemorrhagic stroke, as well as analyzed how these ratios varied among tumor and phenotypic subtypes. In their sub-analysis of 2,613 patients with GH-secreting adenomas, incidence was elevated for both ischemic stroke (standardized incidence ratio = SIR = 1.83) and hemorrhagic stroke (SIR 2.16). When analyzing by ensuing mortality instead of incidence, ischemic and hemorrhagic stroke again occurred at increased frequency compared to the healthy population (hazard ratio = HR = 1.38 and 1.37, respectively).

### CeVD in PA patients with Cushing disease

While less commonly studied than acromegaly, elevated CeVD risk has also been found in patients with Cushing disease (CD). In addition to multiple case studies reporting strokes in patients with CD (32–34), there have been five population-based studies examining the risk of CeVD in patients with some form of Cushing syndrome (CS) (**Table 2**). In 2011, Bolland et al. studied 253 patients with CS, of whom 74.3% were confirmed to have CD (35). While all-cause SMRs were elevated for patients with CD (3.5 for macroadenomas, 3.2 for microadenomas), mortality and incidence ratios were not computed for CeVD. However, they did find that stroke was tied with sepsis as the #2 cause of death in their cohort at 17% of all deaths, behind ischemic heart disease at 19%. Two years later, Dekkers et al. published a nationwide study from Denmark utilizing national datasets to uncover 343 CS patients (211 with CD) (36). By comparing these cases to 34,300 control patients from the same patient registries, they adjusted for age, sex, calendar time, cancer, diabetes, hypertension, COPD, liver disease, and alcohol-related disease, then calculated patient risk for stroke across multiple timepoints. Their study revealed a stroke hazard ratio of 4.5 for patients 3 years before a diagnosis was made, and 4.3 for the first year after diagnosis. On long term follow-up, they found that this risk decreased but did not disappear up to 30 years after diagnosis (HR = 2.1). Dekkers et al. also computed a CD-specific stroke hazard ratio of 2.1 across the same 30 years, but did not find this to be significantly different from the hazard ratio of CS.

**Table 2:** Cerebrovascular outcomes related to PA patients with cushing disease.

Authors and Year	No. of Patients	Population Examined	Main Findings
Bolland et al., 2011 (35)	253	Cushing syndrome; 188 (74.3%) with Cushing disease	17% of patient deaths were due to stroke (#2 cause of death)
Dekkers et al., 2013 (36)	343	Cushing syndrome; 211 (61.5%) with Cushing disease	Stroke HR 3 years before diagnosis: 4.5 Stroke HR in first year after diagnosis: 4.3 Stroke HR 1-30 years after diagnosis: 1.5 Stroke HR for CD 0-30 years after diagnosis: 2.1 Stroke HR for CD was not significantly different from those with adrenal Cushing syndrome
Clayton et al., 2016 (37)	320	Cushing disease	1.9% of patients died from stroke Overall SMR for circulatory disease (CVD and CeVD): 2.17
Papakokkinou et al., 2020 (38)	502	Cushing disease	Overall stroke SMR: 3.0 Stroke SIR for patients in remission: 2.6 Stroke SIR for patients not in remission: 8.2 Stroke SIR from diagnosis to 1 year after remission: 4.9 Stroke SIR > 1 year after remission: 3.1
Oh et al., 2021 (20)	31,983; 19,416 NFPAs (4,077 with hypopituitarism); 8,871 prolactinomas; 2,613 GH-secreting adenomas; 1,083 ACTH/TSH-secreting adenomas.	All PAs	Ischemic stroke SIR in ACTH and TSH-secreting adenomas: 3.75 Ischemic stroke HR in ACTH/TSH adenomas: 1.9 Hemorrhagic stroke SIR in ACTH/TSH adenomas: 6.9 Hemorrhagic stroke HR in ACTH/TSH adenomas: 1.9

HR, hazard ratio; CeVD, cerebrovascular disease; SMR, standardized mortality ratio; CD, Cushing disease; SIR, standardized incidence ratio.

Following Dekkers et al, two more studies were released that briefly examined CeVD: in 2016, Clayton et al. reviewed 320 patients with confirmed CD across four countries and obtained 10 years of monitoring data after their subsequent cure (37). They found an all-cause SMR of 1.61 for these patients, and while SMR for circulatory disease was found to be 2.17, they did not distinguish between cardiovascular and cerebrovascular deaths. In 2020, Papakokkinou et al. conducted a larger Swedish study of 502 patients with CD and did examine CeVD, finding a stroke SMR of 3.0 (38). This was a follow-up study from a publication one year prior on the same database, where cardiovascular disease was found to be the #1 cause of death in patients with CD (39).

While Oh et al. is again the most recent and largest study of CeVD in patients with CD, its CD analysis suffers from the fact that the study combined 1,083 ACTH and TSH-secreting adenomas into one cohort (20). In this combined population, they found an SIR of 3.75 for ischemic stroke, an SIR of 6.9 for hemorrhagic stroke, and HRs of 1.9 for both. In spite of the limitation of combining these cohorts together, Oh et al. also notably reports that this group had the highest rates and risk for stroke among all adenoma groups examined.

### CeVD in other PA subpopulations

Studies examining CeVD in non-acromegalic non-Cushingoid PA patients are less common and utilize differing patient subpopulations for analysis (**Table 3**). In a general study of all PA patients, Brada et al. examined a cohort of all PAs in 1999, and found an elevated risk for CVAs compared to the healthy population (RR 4.1) (19). Two other studies examined CeVD in patients with hypopituitarism, which largely consisted of patients with PAs. Bülow et al. examined 344 patients who had at least one deficient pituitary axis, regardless of which hormone(s) was/were deficient (40). While their cohort included patients with other causes of hypopituitarism, PAs were responsible in 88% of the patients. Patients with acromegaly and Cushing disease were excluded, as were patients on GH replacement therapy and those who died within 1 month of pituitary surgery. Among the remaining patients, CeVD mortality was significantly elevated (SMR 3.39), and CeVD was found to be the top cause of death among patients who died from cardiovascular disease. In a similar study with a larger population, Tomlinson et al. also examined patients with at least one degree of hypopituitarism in 2001 (41). In their cohort of 1,014 patients, 57% had NFPAs and 9% had prolactinomas, while the most common non-PA cause was craniopharyngioma at 12%. Acromegalic and Cushing disease patients were again excluded, as were patients with metastases to the pituitary. They reported a standardized mortality ratio for CeVD of 2.44, a finding that persisted when narrowing analysis to NFPAs. When comparing adenoma subtypes, however, SMR did not vary by a specific hormone deficiency or by the number of deficient endocrine axes.

**Table 3:** Cerebrovascular outcomes related to PA patients without acromegaly or cushing disease.

Authors and Year	No. of Patients	Population Examined	Main Findings
Bülow et al., 1997 (40)	344	Patients with ≥1 degree of hypopituitarism from treatment for prior tumor (PA 88%, CP 12%). Patients with acromegaly or Cushing disease and those on GH replacement therapy were excluded. Deaths within 1 month of surgery were excluded.	Overall CeVD SMR: 3.39. CeVD was the top cause of death among cardiovascular deaths. CeVD mortality was independent of duration of pituitary insufficiency CeVD mortality was greater in patients younger than 55 at time of diagnosis (SMR 6.67). Age-related SMR differences were not found for cardiovascular mortality.
Brada et al., 1999 (19)	331	PAs (63% NFPA) after undergoing any form of treatment.	64 patients (19.3%) suffered a CVA. Risk of CVA was higher in patients with PAs (RR 4.1). Tumor type did not affect CVA risk (except acromegaly).
Tomlinson et al., 2001 (41)	1,014	Patients with ≥1 degree of hypopituitarism, regardless of hormone (57% NFPA, 12% CP, 9% prolactinoma, 9% idiopathic hypopituitarism). Patients with acromegaly, Cushing disease, or metastases to the pituitary were excluded.	23 patients (2.3%) died from CeVD. Overall CeVD SMR 2.44. NFPA diagnosis carried a CeVD SMR of 2.44. Overall SMR was not correlated with specific hormone deficiencies, or number of deficient endocrine axes.
Olsson et al., 2015 (42)	2,795	NFPAs (54% with hypopituitarism)	Overall CeVD SMR: 1.73. Ischemic heart disease mortality was not elevated relative to healthy populations.
Olsson et al., 2016 (43)	2,795	NFPAs (54% with hypopituitarism)	Cerebral infarct SIR 1.66. SIR in young adults with NFPAs: 5.75. Hypopituitarism significantly increased stroke incidence in women, but not in men.
Oh et al., 2021 (20)	31,983; 19,416 NFPAs (4,077 with hypopituitarism); 8,871 prolactinomas; 2,613 GH-secreting adenomas; 1,083 ACTH/TSH-secreting adenomas.	All PAs	Ischemic stroke SIR in ACTH and TSH-secreting adenomas: 3.75 SIR in NFPAs without hypopituitarism: 3.12 SIR NFPAs with hypopituitarism: 3.1 SIR in prolactinomas: 2.9 SIR in GH adenomas: 1.8 Ischemic stroke HR in ACTH/TSH adenomas: 1.9 HR in NFPAs with hypopituitarism: 1.9 HR in NFPAs without hypopituitarism: 1.6 HR in GH adenomas: 1.38 HR in prolactinomas: 1 Hemorrhagic stroke SIR in ACTH/TSH adenomas: 6.9 SIR in NFPAs with hypopituitarism: 5.4 SIR in NFPAs without hypopituitarism: 4.0 SIR in prolactinomas: 3.9 SIR in GH adenomas: 2.2 HR in ACTH/TSH adenomas: 1.9 HR in NFPAs with hypopituitarism: 1.9 HR in NFPAs without hypopituitarism: 1.7 HR in GH adenomas: 1.37 HR in prolactinomas: 1 Among NFPAs, the effects of hypopituitarism were not statistically compared. 95% CIs show hypopituitarism increased SIR for hemorrhagic stroke but not for ischemic stroke.

CP, Craniopharyngioma; CeVD, cerebrovascular disease; SMR, standardized mortality ratio; GH, growth hormone; SIR, standardized incidence ratio; HR, hazard ratio; CI, confidence interval.

While two studies narrowed their inclusion criteria to only study nonfunctional adenomas, they were based on the same cohort. Olsson et al. published studies in 2015 and 2016 on 2,795 PAs. In 2015 their study found an SMR of 1.73 for CeVD, while their 2016 study found a standardized incidence ratio of 1.66 for cerebral infarct. Hypopituitarism was present in 54% of these patients, but due to the nature of using a national registry for data collection, degree of hypopituitarism, specific hormone deficits, and the role of hormone replacement therapy could not be analyzed.

Similar to acromegaly and Cushing disease, the most comprehensive study on other PAs is Oh et al. from 2021 (20). Their cohort of 31,983 patients included 19,416 NFPAs, 8,871 prolactinomas, 2,613 GH-secreting adenomas, and 1,083 ACTH or TSH-secreting adenomas. Among their NFPAs, 4,077 patients had documented hypopituitarism. The authors examined the SIR for both ischemic and hemorrhagic stroke, as well as analyzed how these ratios varied among tumor and phenotypic subtypes.

For ischemic stroke, Oh et al. found an overall SIR of 3.0 and hazard ratio of 2.2. When examining by tumor type, the highest SIR was uncovered among patients with ACTH and TSH-secreting adenomas (SIR 3.75), followed by NFPAs without hypopituitarism (3.12), NFPAs with hypopituitarism (3.1), prolactinomas (2.9), and lastly GH subtypes (1.8). Hazard ratios for ischemic stroke were similarly highest for ACTH/TSH adenomas (1.9). Following these, NFPAs with (1.9) and without (1.6) hypopituitarism followed, with GH subtypes after them (1.38). Prolactinomas carried a hazard ratio of 1 for ischemic stroke.

When examining hemorrhagic stroke, Oh et al. found an SIR of 4.2 and hazard ratio of 2.8. The highest SIR was again found for ACTH/TSH adenomas (6.9), followed by NFPAs with (5.4) and without (4.0) hypopituitarism. Prolactinomas were fourth with a SIR of 3.9, and GH subtypes carried the lowest SIR of 2.2. For hazard ratios, ACTH/TSH subtypes and NFPAs with hypopituitarism again carried the highest risk (1.9), with non-hypopituitarism NFPAs following (HR 1.7), and GH adenomas last (1.37). Again, prolactinomas carried a hazard ratio of 1.

### CeVD in PA patients by patient sex

Due to the sex-dependent function of the hypothalamic-pituitary axis, many of the studies examining CeVD among PA patients report sex-based morbidity and mortality (**Table 4**). Wright et al’s study on acromegaly found that CeVD mortality was significantly elevated in women but not men (23). In 2016, Olsson et al’s second study on 2,795 NFPAs found a greater increase in cerebral infarction incidence in women (SIR 2.28) compared to men (SIR 1.32; P < 0.0001) (43). But while many other studies also report CeVD outcomes by patient sex, direct comparisons between sexes are either omitted from analysis or yield nonsignificant results. While Bülow et al’s 1997 study of patients with hypopituitarism reports a higher SMR for CeVD mortality in women (4.91) compared to men (2.64), no statistical test is reported, and 95% confidence intervals of SMR overlap (women: 2.62-8.40; men: 1.44-4.42) (40). Olsson et al’s prior NFPA study in 2015 reports higher overall SMR for women than men, but differences in cerebrovascular mortality were nonsignificant (42). Brada et al’s 1999 study on all PAs found a higher risk of CVAs among women (RR 6.1) compared to men (1.68), but this sex-based difference was rendered nonsignificant on multivariable analysis (19). And lastly, in Oh et al’s national study on almost 32,000 PAs, sex differences in stroke incidence were not examined, and 95% confidence intervals of sex-based SIRs overlap (20).

**Table 4:** Cerebrovascular outcomes related to PA patient sex.

Authors and Year	No. of Patients	Population Examined	Main Findings
Wright et al., 1970 (23)	194	Acromegaly	CeVD mortality was significantly elevated in women but not in men.
Bülow et al., 1997 (40)	344	Patients with hypopituitarism from treatment for prior tumor (PA 88%, CP 12%). Patients with acromegaly or Cushing disease and those on GH replacement therapy were excluded. Deaths within 1 month of surgery were excluded.	CeVD SMR in women: 4.91 CeVD SMR in men: 2.64 Sex-based differences in SMR are nonsignificant.
Brada et al., 1999 (19)	331	PAs (63% NFPA) after undergoing any form of treatment.	CVA RR in women with PAs: 6.1 CVA RR in men with PAs: 2.9 Sex-based differences in RR are nonsignificant on multivariate analysis.
Olsson et al., 2015 (42)	2,795	NFPAs	CeVD SMR in women: 1.79 CeVD SMR in men: 1.68 Sex-based differences in SMR are nonsignificant
Olsson et al., 2016 (43)	2,795	NFPAs	Cerebral infarction SIR in women: 2.28 Cerebral infarction SIR in men: 1.32 Sex-based difference is significant Presence of hypopituitarism significantly increased SIR in women, but not in men.
Oh et al., 2021 (20)	31,983; 19,416 NFPAs (4,077 with hypopituitarism); 8,871 prolactinomas; 2,613 GH-secreting adenomas; 1,083 ACTH/TSH-secreting adenomas.	All PAs	Overall ischemic stroke SIR: 3.03 Overall hemorrhagic stroke SIR: 4.15 95% Cis show sex-based differences in SIR are nonsignificant. Ischemic stroke mortality HR in women: 0.63. Hemorrhagic stroke mortality HR in women: 0.62.

CeVD, cerebrovascular disease; CP, Craniopharyngioma; SMR, standardized mortality ratio; RR, risk ratio; GH, growth hormone; SIR, standardized incidence ratio; HR, hazard ratio.

## Proposed mechanisms of CeVD in PA patients and effect of PA treatment on CeVD

Cerebrovascular disease poses an excess risk of morbidity and mortality among patients with PAs. Epidemiological studies on this risk select differing cohorts for analysis, either examining all PAs, patients with acromegaly, those with Cushing disease, NFPAs only, or patients with some degree of hypopituitarism. Each study confirms the elevated rate of stroke or death from overall cerebrovascular disease but the mechanisms behind the elevated risk remain debated. The greatest amount of evidence exists in the fields of acromegaly and Cushing disease, while the pathophysiology behind elevated CeVD risk in other PAs remains less well understood.

### Patients with acromegaly: Proposed mechanisms of CeVD and impact of treatment on CeVD

Clinical studies have shown that patients with acromegaly suffer from increased rates of cerebrovascular disease and death (**Table 1**). The high rate of CeVD leads to increased incidence of ischemic (SIR 1.83) and hemorrhagic (SIR: 2.16) strokes (20), and among the literature CeVD is responsible for 2.4-15.4% of deaths in acromegalic patients. This evidence highlights the need for continued CeVD monitoring among these patients, and a high index of suspicion for CeVD should be maintained for patients with uncontrolled or recently diagnosed acromegaly.

Elevated CeVD risk in acromegalic patients may stem from excess GH secretions. Elevated GH has been shown to enhance atherosclerotic disease by causing cardiomyopathy, insulin resistance, hypertension, and lipid alterations (44). It is likely that this effect stems from increased downstream IGF-1 levels, which causes smooth muscle hyperplasia in early atherosclerosis (45). One study found that increased GH elevates plasma triglycerides and decreases HDL, and that normalization of GH and IGF-1 will restore triglycerides and HDL to a normal level (46). These findings of GH and IGF-1 induced insulin resistance, triglyceride and lipid alterations are sure to influence a patient’s risk of vasoocclusion through the effects of cumulative atherosclerotic disease. However, elevated GH and IGF-1 may also cause structural changes and degradation of blood vessels in a more direct manner as well. Andersson et al. found that relative to controls, transgenic mice overexpressing bovine GH displayed impairment in carotid artery relaxation and poor vascular endothelial cell function (47). These findings underscore the complex and multi-faceted impact that excess GH may have on acromegalic patients’ cerebrovasculature.

While these molecular and animal studies show how GH and IGF-1 may influence the entire circulatory system, the clinical research above shows how its effect on CeVD risk is quite different from that of cardiovascular disease. Orme et al’s 1998 UK study on 1,362 acromegalic patients examined how GH levels, duration of acromegaly, and age at diagnosis affected mortality (27), and found near opposite effects on cardiovascular and cerebrovascular risk profiles; while duration of GH excess positively correlated with CeVD mortality, cardiovascular mortality was not affected. While low post-treatment serum GH levels reduced cardiovascular mortality, it did not affect CeVD mortality. And while cardiovascular mortality was not age-dependent, a younger diagnosis of acromegaly further elevated CeVD Mortality. Together, the findings of Orme et al. suggest that the cerebrovascular network may be more fragile and less dynamic than the cardiovascular system, and as a result, long durations of even slightly elevated GH can cause irreversible damage. These findings are supported by Andersson et al’s transgenic mouse experiment reported above, where endothelial cell deterioration in mice overexpressing GH was found to be vessel location-specific (47). The fact that Orme’s younger patients displayed higher elevations of CeVD mortality further suggests that this damage leads to structural changes within the cerebrovascular network that continue to accumulate CeVD risk in the years following acromegaly treatment.

While successful treatment of acromegaly will normalize GH and IGF-1 levels, it remains unclear if CeVD risk normalizes after achieving biochemical remission in these patients. The initial treatment for patients with PAs causing acromegaly is surgery, followed by radiation therapy and/or medical management for patients failing to achieve biochemical remission, and long-term management of serum triglyceride, lipid, and glucose concentrations. Recently Ayuk and Sheppard found that low levels of serum GH after treatment have been correlated with reduced overall cardiovascular disease mortality, reporting that achieving a post-treatment GH level of less than 2.5 ug/L can restore overall SMR to a normal level (48). These findings have been corroborated by other studies that found the 2.5 ug/L threshold to be somewhat arbitrary, as drawing lower and lower thresholds further reduced overall SMR (28, 49). Indeed, as treatment strategies for acromegaly have been refined over the decades, overall long-term mortality has declined as a result (31). However despite these advances in overall care and outcomes, cerebrovascular disease may remain an exception to this trend. Orme et al’s 1998 study found that CeVD mortality remained elevated in treated patients, and standardized mortality ratio was not affected by post-treatment GH levels (27). While early intervention will likely help lower cumulative CeVD risk, a delay in diagnosis and management may lead to irreversible CeVD risk increase. Thus, successful reduction of lifetime CeVD risk in patients with acromegaly currently depends on prompt treatment after diagnosis, composed of both tumor control and medical therapy to normalize the many metabolic factors altered by abnormal GH and IFG-1 levels. Without addressing both the tumor and its metabolic sequelae, patients may accumulate risk for future atherosclerotic disease and ensuing CeVD.

### Patients with CD: Proposed mechanisms of CeVD and impact of treatment on CeVD

Similar to acromegaly, clinical studies have shown the increased rate of stroke among patients with CD (**Table 2**). While each study does include CD patients, several publications are limited by the inclusion of patients with any form of Cushing syndrome or by combining CD patients with other adenoma types (20, 35, 36). However, while this means the uncovered mortality rates may not be exact representations of patients with ACTH-secreting pituitary adenomas, they nevertheless demonstrate a stark reality that those with CD experience elevated levels of cerebrovascular morbidity and mortality.

CeVD in patients with Cushing disease is most likely a result of the wide variety of metabolic derangements caused by Cushing syndrome. Excess glucocorticoids lead to induction of gluconeogenesis and disruption of insulin receptor signaling, while also increasing expression of adipose triglyceride lipase and hormone sensitive lipase, which control lipid breakdown in adipocytes and increase circulating free fatty acid levels (50, 51). Cortisol is also known to affect the coagulation system, arterial wall stiffness, and heart function as well, and altogether these metabolic changes manifest in many Cushingoid symptoms that affect cerebrovascular disease risk: central fat redistribution, hypertension, impairment of glucose tolerance, hyperlipidemia, and hypercoagulability (36, 38, 50, 52, 53). These changes are found in more specific studies of CD as well, where patients have been demonstrated to have higher BMI, waist:hip ratio, systolic and diastolic blood pressures, fasting glucose and insulin levels, total cholesterol, low density lipoprotein, fibrinogen, and lipoprotein A (52).

Metabolic changes in CD also impact cerebrovascular disease risk by directly altering patient vasculature. On ultrasound studies of patients with CD, atherosclerotic plaques were found in 26.7% of patients, while zero were found in age and sex-matched controls (52). Patient vessels also demonstrated increased intima-media thickness and higher vessel peak flow velocities, while lumen diameters and distensibilities both decreased (52). Another study found that the cross-sectional area of tunica media within small-resistance arteries is significantly greater in CS patients compared to hypertensive patients and controls (54). These vascular changes are likely multi-mechanistic; glucocorticoid excess can lead to activation of mineralocorticoid receptors and cause small-vessel fibrosis (55), and animal and human studies have shown accelerated atherosclerosis after prolonged corticosteroid exposure (56, 57). However, it has also been suggested that this vascular change is caused by an increased level of oxidative damage, as CS patients have been found to have increased oxidative stress markers and lower antioxidant markers (58). Thirdly, studies in untreated CD and CS patients have found elevated endothelin-1 levels relative to controls (59). These levels were similar between patients with CD and CS, were even further elevated in the three patients who died, and were correlated with total cholesterol levels in patients as well. The combined effect on vessels from these multiple sources results in increased damage and remodeling in CS patients, even when comparing to controls with similar levels of hypertension (60).

Increased glucocorticoid levels are also responsible for hypertension in Cushing syndrome and disease, which is seen in 55-85% of all adults and half of all children with CS (50, 61). Glucocorticoids inhibit vasodilatory actions by nitric oxide synthase, prostacyclin, and kinin-kallikrein, activate the renin-angiotensin system, increase vascular reactivity to vasoconstricting catecholamines, and inhibit peripheral catabolism of norepinephrine (53). Increased levels of cortisol may also exceed the catabolism capabilities of 11-beta-hydroxysteroid dehydrogenase type 2, allowing the remaining molecules to bind to mineralocorticoid receptors and behave like aldosterone as well (53). In this multi-mechanistic setting of Cushing induced hypertension, already-present atherosclerosis is likely exacerbated, and treatment with antihypertensive medications may not help until normal glucocorticoid levels are restored in patients.

In summary, glucocorticoid excess affects CeVD risk in CD patients by negatively impacting metabolic factor homeostasis, and directly affects patient vasculature by increasing rates of atherosclerosis and hypertension. The role of glucocorticoids, rather than ACTH, as the main causative factor of CeVD risk, is further supported by the fact that studies examining CD and CS found similar risk levels, and that risk is significantly elevated in pre-diagnostic cohorts where disease is uncontrolled (**Table 2**). But in addition to these patients with severe or uncontrolled disease, cohorts that examine pre-diagnosis timelines also include patients with subclinical CD and CS as well. These patients are also likely at risk for increased rates of CeVD, as one study found that adrenal adenoma patients with subclinical levels of excess cortisol experienced higher rates of metabolic syndrome, diabetes mellitus, and previous cerebrovascular events than patients with nonfunctional adrenal adenomas (62). These metabolic and vascular changes associated with CD significantly increase risk for CeVD, even in mild cases.

Similar to acromegaly, the mainstay of treatment in CD is a multifaceted approach of transsphenoidal tumor resection and radiation for tumor control followed by medical management of residual hormonal and metabolic sequelae. Unfortunately, also similar to acromegaly, it appears that while successful tumor and metabolite management may prevent further accumulation of cerebrovascular risk, it may not reverse damage already sustained by cerebral vasculature. Dekkers et al’s 2013 study on CS patients found persistently elevated stroke rates even when specifically analyzing follow-up data after diagnosis and intervention (CD Stroke HR for 30 years of follow-up = 2.1). Risk levels were similar to what was seen in CS patients (30 year HR = 1.5). While not all treatments are effective, and hypertension can often persist after treatment for CS (61, 63), Dekkers et al. found this elevated morbidity and mortality was present in patients with and without persistent disease after surgical intervention. These findings were further confirmed by Papakokkinou et al. in 2020, which analyzed 10 years of follow up data for CD patients in remission (38). In addition to uncovering an overall stroke SIR of 8.2 for patients not in CD remission, they also found increased stroke incidence in patients who had been in remission for over one year (SIR 3.1). While future studies may uncover new ways to reverse the damage incurred by years of untreated CD, the best method of reducing CeVD risk in CD patients is early intervention, tumor control, and restoration of metabolic and vascular homeostasis.

### Other PA patients: Proposed mechanisms of CeVD and impact of treatment on CeVD

Similar to acromegaly and Cushing disease, sufficient clinical evidence has shown that patients with other PA subtypes also suffer from an increased risk of cerebrovascular disease (**Table 3**). But while the causes of CeVD in acromegaly and Cushing disease are well-understood, less is known about the mechanisms behind CeVD risk in other PAs. For example, while Oh et al. found the highest risk of CeVD incidence and mortality in TSH and ACTH-secreting adenomas (20), their study appears to be the first of its kind in examining the cerebrovascular morbidity and mortality in TSH-secreting adenomas. While a likely explanation for this elevated risk includes uncontrolled hypertension secondary to hyperthyroidism, and while treatment *via* tumor removal plus thyroid hormone and blood pressure control may help ameliorate this risk, further research is essential in better defining the underlying mechanisms as well as the long-term effect of treatment on CeVD risk in these patients.

One likely cause of increased CeVD mortality stems from PAs that cause growth hormone deficiency (GHD); like GH excess, GH and IGF-1 deficiency have been shown to enhance atherosclerotic processes and facilitate cardiovascular morbidity and mortality, causing growth hormone homeostasis to resemble a “goldilocks effect” where balance is critical (44, 45, 64, 65). Lewis dwarf rat studies have shown that GHD increases vascular oxidative stress, promoting adverse vessel structure that manifests as late-life stroke and reduction of rat lifespan (66). This hypothesis is supported by clinical evidence as well, where it has been shown that GHD can cause abnormal body composition, reduced lean body mass/increased fat mass, high waist:hip ratio, insulin resistance, lipid abnormalities, and vascular endothelial dysfunction (67–70). Velhelst et al. found that GHD duration was tied to metabolic syndrome, which they found to be associated with higher CeVD morbidity (prevalence ratio of 1.77 relative to patients without metabolic syndrome) (71). In another cohort study, the increased intima-media thickness caused by low GH was seen in children, providing further support that GHD increases cardiovascular risk even in the absence of traditional atherosclerotic risk factors (72). Physicians should remain vigilant for signs of CeVD in GHD patients in all ages, especially so in those without access to GH therapy. Due to the high cost of medical treatment as well as the perception by some insurers that GH is not needed in older populations, uninsured patients or the elderly may be at increased risk for lack of sufficient therapy.

In addition to GHD enhancing atherosclerosis, it may also influence post-stroke outcomes. Adequate GH and IGF-1 is needed to facilitate extracellular matrix remodeling and angiogenesis, and both hormones have been shown to exhibit neuroprotective effects on ischemic cells of the central nervous system (45, 73–76). It is believed this neuroprotection stems from vascular endothelium acting as an autocrine and paracrine gland in ischemic conditions, releasing factors and hormones including GH that prevent cell necrosis and stimulate nearby arteriogenesis (76). Recent animal studies have supported this hypothesis and demonstrated how GH administration in mice exposed to cerebral infarct improves motor function and recovery by 50-60% (77). If GH and IGF-1 are indeed such critical hormones for cerebral vessel recovery after cerebrovascular infarct, it would come as no surprise that GHD patients who suffer from stroke might also experience poorer outcomes.

While this quantity of evidence means GHD and ensuing IGF-1 deficiency is likely a risk factor for CeVD mortality in patients with PAs, it is just as likely that other factors in PA patients are at work as well. Most research has been devoted to growth hormone’s effects on vessel structure and function, but patients with isolated prolactinomas, Cushing disease, or hypothyroidism have also been shown to experience increases in total cholesterol and LDL, independent from the function of other pituitary axes (46). These nonspecific molecular findings are supported by clinical data as well; Tomlinson et al’s 2001 study on 1,014 patients with hypopituitarism (66% of which were due to PAs) found a SMR for CeVD mortality of 2.44 in all patients, but did not find a variation based on specific hormone deficiencies, or on the number of deficient hormone axes (41). While this may be explained by the fact that 70% of hypopituitary patients exhibit some degree of GHD (95% if patients have two or more hormone deficiencies) (78), it has also been shown that hypopituitarism is not the only cause of CeVD risk in PA patients; Olsson et al’s 2016 study on NFPAs found that among the males in their cohort, the elevated stroke incidence seen was the same between patients with and without hypopituitarism (overall SIR 1.66) (43). Oh et al’s study found the highest rate of stroke to be in ACTH and TSH-secreting adenomas, and showed how among NFPA patients with and without hypopituitarism, ischemic stroke incidence did not vary (SIR 3.1) (20). While this means that hormone function and tumor type likely does play a role in CeVD risk, it also shows that the mere presence of PAs increases risk of CeVD and ensuing mortality. The mechanisms behind this risk are likely multifactorial and remain to be fully uncovered, but one such mechanism is likely related to the risk of pituitary apoplexy, which occurs in 0.6-10% of PA cases (79, 80). Select case series have reported cases of stroke caused by apoplexy-induced PA enlargement and compression of the internal carotid arteries, with near-complete resolution of symptoms after emergent tumor debulking (81–84).

Similar to acromegaly and Cushing disease, it is unclear how much effect treatment may or may not have on CeVD risk reduction. After tumors are resected, hormone deficits are treated with restoration of hormone homeostasis *via* supplementation of corticosteroids, thyroid hormones, sex hormones, or GH (44, 85). GH treatment in GHD patients has shown to have beneficial effects on insulin sensitivity, LDL levels, total cholesterol and triglycerides, fat and lean body mass, and diastolic blood pressure (86, 87). In cases where metabolic factors do not return to normal levels, medical management is essential to prevent further accumulation of atherosclerosis and CeVD risk, similar to the treatment of patients with acromegaly. However, also alike those patients with acromegaly, studies have shown that hormone therapy may not help in returning cerebrovascular mortality to normal levels in patients with GHD or hypopituitarism. In two cohort studies on patients with GHD, CeVD mortality remained elevated in GH-treated patients (88, 89). While these studies examined all causes of GHD and not just PAs, Hammarstrand et al. studied 426 NFPA patients in 2018 and found similar results: standardized incidence of cerebral infarct was elevated in the total patient population, and did not appear to vary by who received GH replacement therapy (65). More research is needed to better define the relationship or lack thereof between hormone replacement and variance of CeVD mortality.

While hormone therapy may not lower CeVD risk in this population, other treatments directly targeting the cereberovasculature may show promise. Holmer et al. studied nonfatal CVAs in GHD patients and found that while GHD patients had a significantly higher rate of nonfatal stroke compared to the overall population, stroke incidence declines over the years following diagnosis (90). While the authors conclude that this reduced risk may have been due to GH therapy, most patients in their cohort were on a host of other cardiovascular drugs as well. Their study is not without its shortcomings for root cause analysis: it makes no direct comparisons between specific drug therapies and CVA risk, roughly 40% of study participants had non-PA causes of GHD, and the study excluded all cases of fatal stroke in their analysis. Nevertheless, their findings show promise for potential avenues to reduce CeVD risk in patients with hormone deficits secondary to PAs.

### Sex-based CeVD differences in PA patients: Proposed mechanisms

While nearly every study that stratified by sex found higher CeVD incidence and mortality in women, these differences may in fact be less significant than they appear. Some clinical studies have indeed shown that women with PAs tend to experience higher rates of CeVD morbidity and mortality (20, 23, 43), but in other studies the risk difference between women and men is unexamined or nonsignificant (19, 40, 42). It has been argued that if an increased risk exists, it is likely from the fact that women often have prior exposure history to sex hormones (44), as hormone therapy may be prescribed for contraception, infertility, oligo- or amenorrhea, or for symptoms of menopause. Estrogens were also once thought to lower the cardiovascular risk profile, and were broadly prescribed in the past to most postmenopausal women. This theory has since been disproven (91), but it has been shown that patients who were given conjugated equine estrogen for heart disease prevention have increased risk for stroke in the following years (92).

Regardless of the role of hormones in CeVD risk for women with PAs, the fact remains that women with PAs also experience tumor-specific CeVD risk. One 2013 review article by Erfurth et al. examined patients with GHD and found that in the years since physicians stopped estrogen therapy to prevent heart disease, all-cause mortality rates in women have declined but not returned to normal (93). They found that for patients with GHD caused by tumors, CeVD was the main cause of increased mortality, and among female patients this mortality persisted after GH replacement. Erfurth’s findings suggest that while prior hormone therapy may be a contributing factor to CeVD mortality among women with PAs, it is likely not the sole cause. Like other studies on patients with PAs, it appears that women experience CeVD risk regardless of hormone levels. More studies are needed that directly analyze CeVD mortality between women and men to determine if a significant difference truly exists and what the underlying mechanisms may be.

## Conclusions

The risk of cerebrovascular morbidity and mortality is significantly elevated in patients with pituitary adenomas. Whether affected by GH-excess, GH-deficiency, or endocrine-normal state, this population experiences permanent compromise to the delicate network of blood vessels throughout the central nervous system. While the mechanisms behind this risk and the effects of treatment remain debated, and more work is needed to better elucidate post-treatment risk estimation, for now the best way to prevent CeVD in patients with pituitary adenomas is to diagnose and intervene early, resecting tumors and treating hormone imbalances where indicated. Until more is known about the effects of treatment on reducing risk or halting its further accumulation, physicians should remain vigilant for early signs of cerebrovascular disease in patients with pituitary adenomas, and keep CeVD high on their differential when PA patients present with neurologic symptoms.
